# DNA quadruplexes as molecular scaffolds for controlled assembly of fluorogens with aggregation-induced emission†
†Electronic supplementary information (ESI) available: Supporting figures and tables. See DOI: 10.1039/c8sc00001h


**DOI:** 10.1039/c8sc00001h

**Published:** 2018-01-29

**Authors:** Longyi Zhu, Jun Zhou, Guohua Xu, Conggang Li, Pinghua Ling, Bin Liu, Huangxian Ju, Jianping Lei

**Affiliations:** a State Key Laboratory of Analytical Chemistry for Life Science , School of Chemistry and Chemical Engineering , Nanjing University , Nanjing 210023 , China . Email: jpl@nju.edu.cn ; Email: hxju@nju.edu.cn; b State Key Laboratory of Magnetic Resonance and Atomic and Molecular Physics , Wuhan Institute of Physics and Mathematics , Chinese Academy of Sciences , Wuhan 430071 , China; c Department of Chemical and Biomolecular Engineering , National University of Singapore , 4 Science Drive 4 , Singapore 117585 , Singapore . Email: cheliub@nus.edu.sg

## Abstract

Aggregation-induced emission (AIE) can be generated due to the restriction of intramolecular motions. The controllable assembly of fluorogens with AIE properties (AIEgens) is able to provide a new opportunity for precise manipulation of fluorescent signal transduction. Here, a tetrapod DNA quadruplex (TP-G4) was designed as a molecular scaffold for assembly and precise modulation of light emission of an oligonucleotide-grafted fluorogen with aggregation-induced emission (Oligo-AIEgen). The Oligo-AIEgen was synthesized by attaching the AIEgen to the 3′-terminus of the oligonucleotide through a dibenzylcyclooctyne mediated coupling reaction. The AIEgen emitted no detectable fluorescence in the context of a double-stranded structure. When hybridized to the parallel-stranded TP-G4, several AIEgens were located in close proximity to generate fluorescence. The fluorescence intensity has been precisely regulated by manipulation of the spacer length between the core structure of the scaffold and AIEgen, as well as by altering the quartet number of the G-quadruplex. Similar control of fluorescence was also demonstrated using tetramolecular and bimolecular i-motif quadruplex structures as the scaffolds. These scaffolds provide a proof of concept on the manipulation of molecular interactions, which forms a universal molecular tool for the design of new biosensing strategies.

## Abstract 2

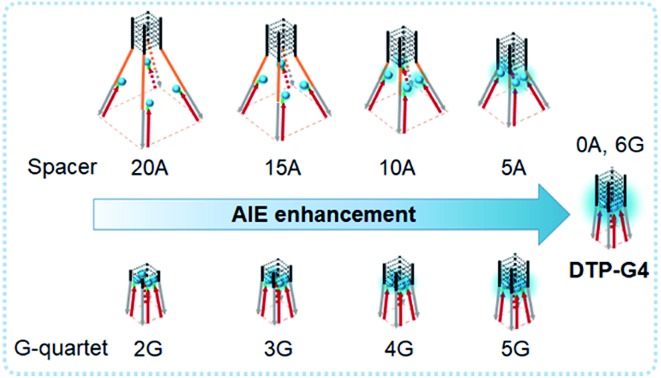
Tetrapod DNA quadruplexes were designed for assembly and precise modulation of light emission of an oligonucleotide-grafted fluorogen with aggregation-induced emission.

## Introduction

Aggregation-induced emission (AIE) is associated with the restriction of intramolecular motions to generate fluorescence. Fluorogens with AIE properties are termed AIEgens. AIEgens have been used in biosensing,^1^–^3^ light-emitting devices,^4^–^7^ and cell imaging^8^–^10^ due to their high brightness in an aggregate state and good photostability. Aggregation of AIEgens can be induced by site-specific reactions,^11^–^13^ analyte binding,^14^–^16^ and changes in environment, such as viscosity and pressure.^17^,^18^ For example, a peptide modified tetraphenylethene (TPE) derivative shows strong AIE upon enzymatic cleavage of a peptide by cathepsin B, which induces the assembly of hydrophobic aggregates.^11^ In another example, the fluorescence of an arginyl-glycyl-aspartic acid (RGD) peptide functionalized with tetraphenylsilole is induced when it is specifically bound to integrin α_v_β_3_ which is used for cell imaging and protein quantification.^14^ As the emission intensities of AIEgens are dependent on the degree of inhibition of intramolecular motions, it is expected that precise control of molecular aggregation will add a new dimension to the fluorescence signal output, which has been difficult to realize so far.

The predictable building blocks of DNA structures make it possible to construct complicated nanostructures.^19^–^21^ DNA can form double- or triple-stranded structures,^22^ inter- or intra-molecular G-quadruplexes^23^–^27^ and i-motif structures,^28^–^31^ which are essential features for the design of molecular scaffolds *in vitro* and *in vivo*.^32^,^33^ Of particular interest are the G-quadruplexes, which were formed by stacking G-quartets of four Hoogsteen-paired guanines. Sequences that are able to adopt G-quadruplex structures are found in 43% of human genes and are prevalent in the promoter regions of oncogenes.^34^ G-quadruplexes have been used as three-dimensional scaffolds to construct DNA assemblies with precisely controlled structures.^35^

**Fig. 1:**
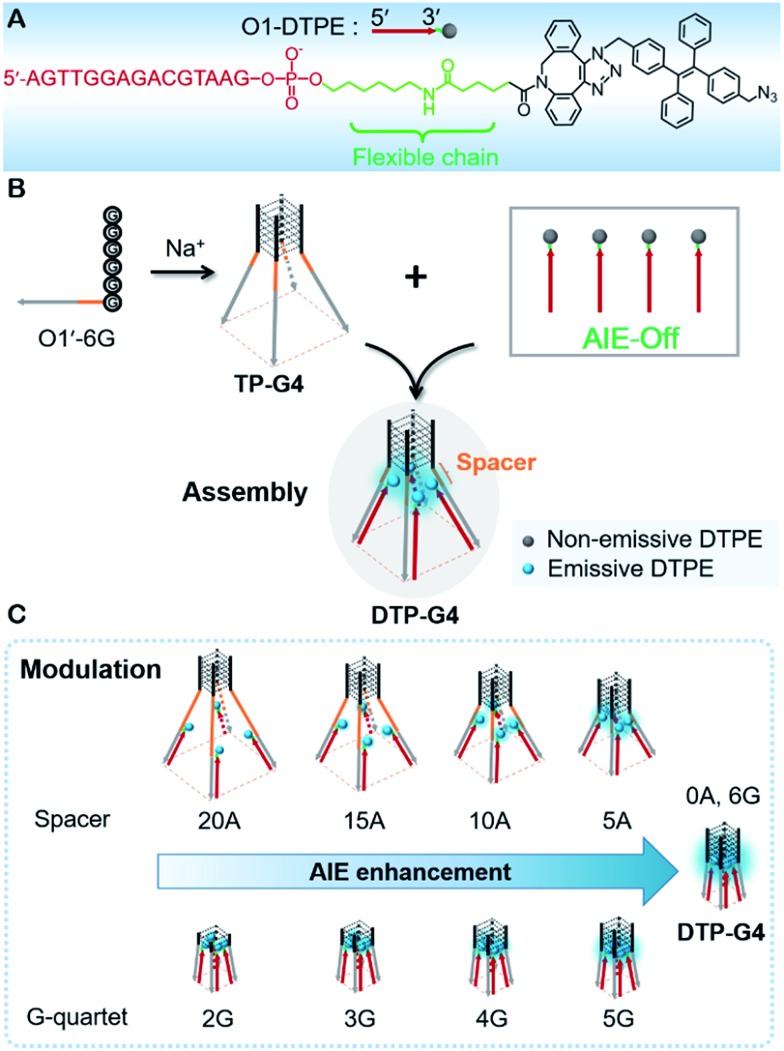
Schematic illustration of (A) the structure of O1-DTPE, (B) tetrapod DNA G-quadruplexes as molecular scaffolds for the assembly of AIEgens and (C) the precise modulation of fluorescence intensity by DNA G-quadruplexes.

Previously, we designed an AIE probe by directly linking TPE to an oligonucleotide. The probe was barely emissive by itself, but the fluorescence was intensified upon the formation of a double-stranded structure,^36^ which restricted the free intramolecular rotation of TPE.^37^ Inspired by the induction mechanism of AIE and the controllability of DNA quadruplex conformation, here we use a tetrapod DNA quadruplex (TP-G4) as a molecular scaffold for the controlled assembly of AIEgens and precise modulation of the fluorescence intensity. To allow free intramolecular rotation of AIEgen in the context of single-stranded DNA and duplex DNA, an alkyl chain of 11 carbon atoms along with a conjugated planar structure was incorporated between the oligonucleotide and AIEgen (Fig. 1A). Upon assembly of the oligonucleotide with the limbs of TP-G4, strong fluorescence was generated due to the localized assembly of AIEgens in the confined space. The precise assembly of the AIEgens was performed by altering the distance between AIEgen and the core structure of the molecular scaffold and by altering the quartet number of the G-quadruplex. Moreover, the oligonucleotide-grafted AIEgen (Oligo-AIEgen) showed a structure-dependent light response to both tetramolecular and bimolecular i-motif quadruplex structures.

## Results

### Design and synthesis of Oligo-AIEgen

**Fig. 2:**
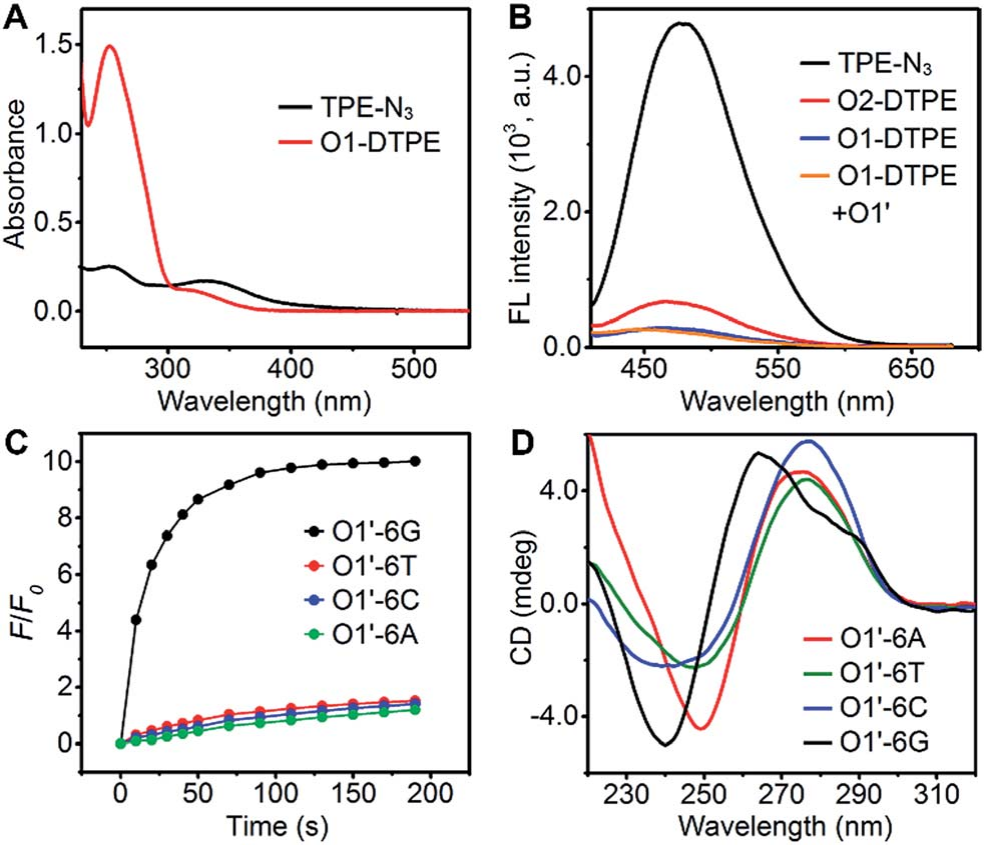
(A) UV absorbance spectra of 10 µM TPE-N_3_ and 10 µM O1-DTPE in DMSO/H_2_O (v/v, 1/399). (B) Fluorescence spectra of 1.0 µM TPE-N_3_, O2-DTPE, O1-DTPE, and the mixture of 1.0 µM O1-DTPE and O1′ in TE reaction buffer at 25 °C. (C) *F*/*F*_0_ of 1.0 µM O1-DTPE in the presence of 1.0 µM O1′-6G, O1′-6A, O1′-6C, and O1′-6T in TE reaction buffer at 25 °C. (D) CD spectra of 20 µM O1′-6G, O1′-6A, O1′-6C, and O1′-6T in TE reaction buffer at 25 °C.

In the synthetic design, a 15 nt oligonucleotide modified at the 3′ terminus with (CH_2_)_6_–NH_2_ (Table S1†) was conjugated with dibenzocyclooctyne-sulfo-*N*-hydroxysuccinimidyl ester (DBCO-sulfo-NHS) through an amide reaction to yield oligonucleotide-grafted DBCO (O1-DBCO). Next, Oligo-AIEgen (O1-DTPE) was prepared by a coupling reaction between bis[4-(azidomethyl)phenyl]-1,2-diphenylethene (TPE-N_3_) and O1-DBCO *via* copper-free click chemistry.^38^ The resulting O1-DTPE was isolated by polyacrylamide gel electrophoresis (PAGE) at a molecular weight of 5558.3 (Fig. S1†). The TPE-N_3_ aggregates in DMSO/H_2_O (v/v, 1/399) showed two UV absorption peaks at 260 and 330 nm. After the click reaction, the absorption at 260 nm was enhanced and slightly blue shifted, indicating the successful synthesis of O1-DTPE (Fig. 2A). The hydrophilic O1-DTPE was water-soluble. Unlike the hydrophobic TPE-N_3_ which aggregated in DMSO/H_2_O (v/v, 1/399) to form particles ranging from 63.5 to 104 nm, the sample of O1-DTPE did not scatter light (Fig. S2†).

TPE-N_3_ clustered into aggregates in DMSO/H_2_O (v/v, 1/399) with strong fluorescence (Fig. 2B).^36^,^37^ As a comparison, the fluorescence intensity of O1-DTPE at 470 nm was greatly weakened to 5.9% of that of TPE-N_3_ in DMSO/H_2_O (v/v, 1/399). We also synthesized O2-DTPE, in which a 44 nt oligonucleotide was conjugated to AIEgen (Table S1†). O2-DTPE shows 2.3-fold higher fluorescence than O1-DTPE because the increase in the nucleotide chain promotes base–base stacking.^39^ Hybridization of O1-DTPE with its fully complementary O1′ does not lead to enhanced fluorescence (Fig. 2B). This is different from the enhanced fluorescence due to duplex formation reported previously for an oligonucleotide conjugated to AIEgen through a shorter alkyl linker.^36^,^37^ These results indicate that molecular flexibility is essential for regulating AIE fluorescence.

### TP-G4 as a molecular scaffold

 The unique structure of the parallel-stranded TP-G4 should provide a molecular-level confined space for the generation of AIE signals (Fig. 1B). To prove this concept, four single-stranded DNA molecules were designed (Table S1†), which consisted of six identical nucleotides, to determine the formation of the G-quadruplex structure and the 15 nt O1′ sequence complementary to the oligonucleotide sequence of O1-DTPE. These strands were named O1′-6G, O1′-6A, O1′-6C and O1′-6T for the number and identity of the 5′ nucleotides (Table S1†).

After O1-DTPE was added to the O1′-6G solution, the fluorescence increased rapidly and reached a plateau in 2 min. The highest signal was 10.4-fold over the baseline. The fluorescence of the solution only slightly increased when O1-DTPE was mixed with O1′ strands with any of the other three nucleotides at the 5′ end (Fig. 2C). Similar phenomena were observed when O2-DTPE was mixed with a 6 nt overhang borne O2′ (Fig. S3†). In addition, the fluorescence intensity increased with the number of O1-DTPE molecules in the presence of O1′-6G (Fig. S4†).

To demonstrate that the fluorescence enhancement observed with O1′-6G and O2′-6G was due to the formation of a G-quadruplex structure, we used native PAGE analysis and circular dichroism (CD) measurements (Fig. 2D). During native PAGE, a high-molecular weight band was observed in the samples of O1′-6G, which was attributed to the tetramolecular structure; high-molecular weight bands were not observed in samples of O1′-6A, O1′-6C, or O1′-6T (Fig. S5†). The CD spectra of O1′-6A, O1′-6C, and O1′-6T have positive peaks, characteristic of single-stranded DNA, at 280 nm. In contrast, the spectrum of O1′-6G has a positive peak at 264 nm and a negative peak at 240 nm (Fig. 2D), indicative of the formation of a parallel G-quadruplex.^24^ The broad peak in the CD spectrum of O1′-6G in the region from 275 nm to 300 nm is likely attributed to the single-stranded O1′ oligonucleotide limbs. The fact that the AIE signal is dependent on the formation of TP-G4 was further shown by the preparation of solutions of O1′-6G and O1-DTPE in a reaction buffer which contains Na^+^, in a buffer containing Li^+^, and in water. Fluorescence was observed only from the buffer containing Na^+^ (Fig. S6†). The G-quadruplex cannot be formed in the presence of Li^+^ ion or without salts.^23^

In order to verify that O1-DTPE could interact specifically with the G-quadruplex scaffold, O1-DTPE was incubated with O1-6G and with 15T-6G (Table S1†). O1-6G and 15T-6G form G-quadruplex structures with limbs that are not complementary to O1. Fluorescence was not observed upon incubation of O1-DTPE with either of these G4 structures (Fig. S7†), indicating that light emission could only be generated upon hybridization of O1-DTPE with the limbs of the clawed structure. Moreover, the sharp fluorescence increase of O1-DTPE was a unique property of TPE, as incubation of any of the O1′ strands with O1-aminomethylcoumarin (O1-AMCA), O1-fluorescein amidite (O1-FAM), or O1-cyanine 3 (O1-Cy3) resulted in only a very slight fluorescence change, whereas the fluorescence intensity of O1-DTPE in the presence of O1′-6G improved by approximately 10-fold that of O1-DTPE in the absence of O1′-6G (Fig. S8†).

### Precise control of light emission from assembled TPE

**Fig. 3:**
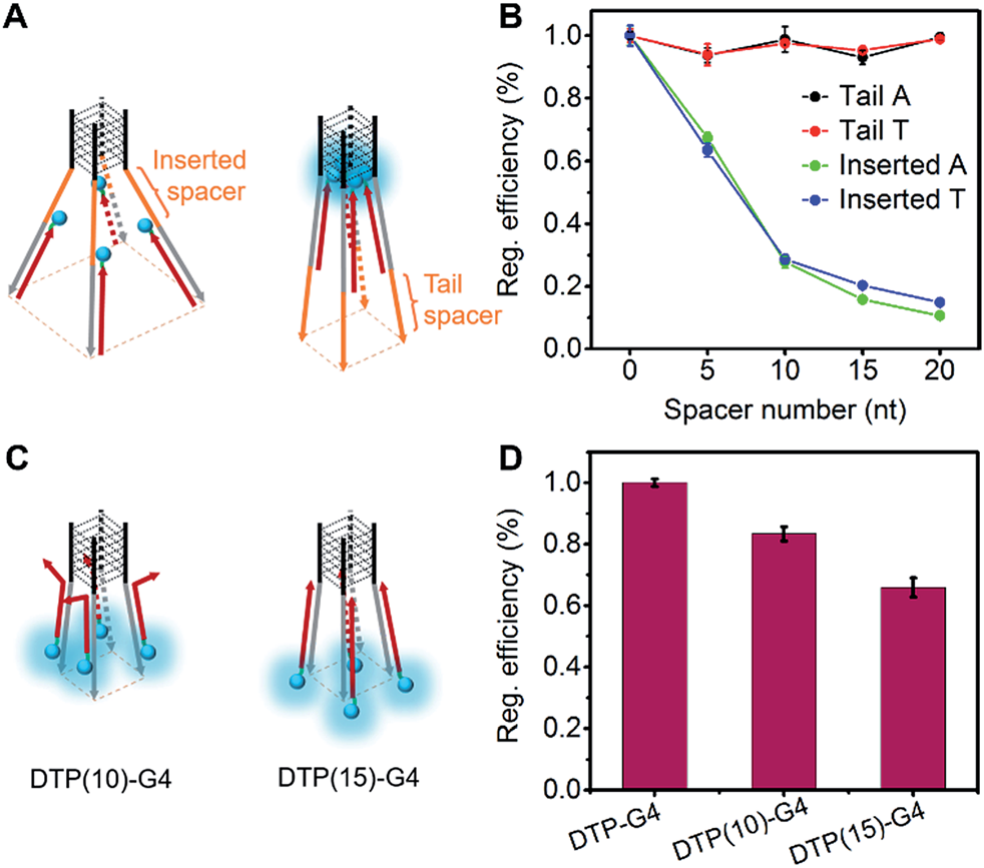
(A) Schematic illustration of locations of spacers relative to G4. (B) Regulation efficiency of DTP-G4 with inserted spacer or tail spacer. (C) Schematic illustration of DTP(10)-G4 and DTP(15)-G4 hybridized with O1″-DTPE. (D) Regulation efficiency of DTP-G4 with partially (10 bp) or fully (15 bp) complementary base pairs to O1″.

To precisely control the molecular interaction and manipulate the light emission, we inserted spacers of different lengths of contiguous A nucleotides between the parallel G-quadruplex and the O1′ limbs (Fig. 3A). Oligonucleotides with spacers of 5, 10, 15, and 20 A nucleotides were synthesized (Table S1†). From the fluorescence intensities, we determined that the regulation efficiency was 100% when O1-DTPE was fully hybridized with TP-G4. Accordingly, O1′-5A-6G, O1′-10A-6G, O1′-15A-6G, and O1′-20A-6G had regulation efficiencies of 67.3%, 27.8%, 15.7% and 10.6%, respectively (Fig. 3B). It is obvious that the fluorescence signal decreased as the fluorophore was located further from the G4 structure, as a result of the separation of the four limbs,^35^,^40^ and less restriction was imposed on the TPE molecules. When different lengths of T nucleotides were used as the spacers (Table S1†), similar reductions in regulation efficiency were observed. A or T bases at the tail of the limb did not significantly alter the regulation efficiency (Fig. 3B).

The light emission modulation protocol was further verified with O1″-DTPE, which has TPE on the 5′ end of the oligonucleotide. When O1″-DTPE was mixed with G4-forming oligonucleotides with the limbs partially (10 bp) or fully (15 bp) complementary to O1″, DTP(10)-G4 and DTP(15)-G4, respectively (Fig. 3C), regulation efficiencies of 84.1% and 67.3% were observed due to the localized assembly of TPE molecules (Fig. 3D). This demonstrated that the AIE signal could be regulated by changing the number of base pairs between AIEgen and TP-G4.

**Fig. 4:**
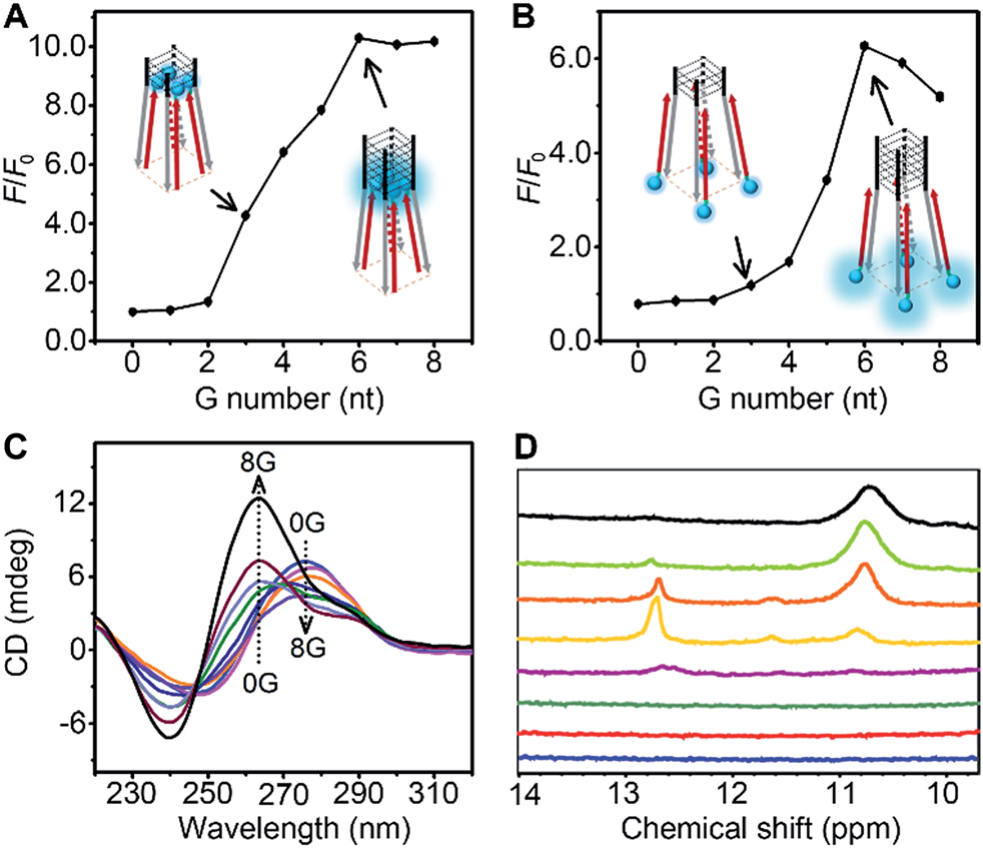
Ratios of fluorescence peak intensity of (A) 1.0 µM O1-DTPE and (B) O1″-DTPE after and before (*F*/*F*_0_) hybridization with O1′-*n*G (*n* = 0 to 8) in TE reaction buffer at 25 °C. (C) CD spectra of 20 µM O1′-*n*G (*n* = 0 to 8) in TE reaction buffer at 25 °C. (D) ^1^H-NMR spectra of O1′-*n*G (*n* = 1 to 8 from bottom to top) in 10 mM phosphate buffer pH 7.4, with 100 mM NaCl and 10 mM MgCl_2_ at 25 °C.

**Fig. 5:**
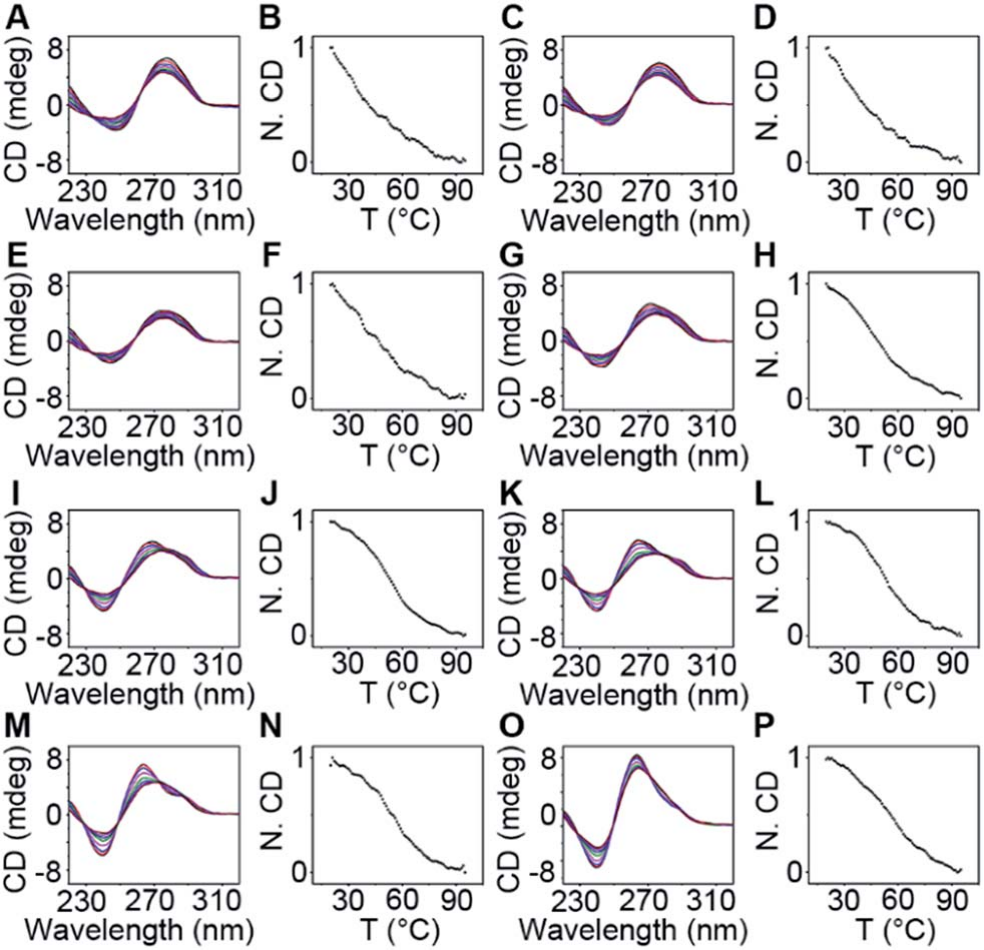
CD spectra at 25, 35, 45, 55, 65, 75, 85, 95 and 99 °C (from top to bottom) at 264 nm, and normalized CD intensities (N. CD) as a function of temperature recorded at 264 nm with the temperature increased by 1.0 °C per 0.5 min of 20 µM (A and B) O1′-1G, (C and D) O1′-2G, (E and F) O1′-3G, (G and H) O1′-4G, (I and J) O1′-5G, (K and L) O1′-6G, (M and N) O1′-7G, and (O and P) O1′-8G. Data are normalized to the highest CD value of 264 nm.

The thermodynamic stability of a parallel G-quadruplex is dependent on the number of G quartets. Therefore, the light emission process could theoretically be controlled by altering the G quartet number. We synthesized O1′ strands with 0 to 8 G nucleotides at the 5′ end. As the number of G nucleotides increased, the fluorescence signals of both O1-DTPE and O1″-DTPE sharply increased and then reached a plateau at 10.4 and 6.2-fold over the baseline, respectively (Fig. 4A and B). This indicated that the fluorescence correlated initially with the G-quadruplex structure stability as demonstrated by CD and melting temperature measurements with complete sigmoidal curves for oligonucleotides with 4 to 8 G nucleotides (Fig. 5). The CD spectra of O1′-0G, O1′-1G, and O1′-2G did not show a peak characteristic of a G4 structure, but strong CD peaks attributed to single-stranded DNA were observed at around 280 nm. Peaks characteristic of G4 (a positive peak at 264 nm and a negative peak at 240 nm) were observed in the CD spectra for oligonucleotides with 3 to 8 G nucleotides, with absolute peak intensities associated with G4 gradually increasing and the intensities of the peaks at 280 nm decreasing with the increasing G number (Fig. 4C). The melting temperature reached a plateau for O1′-6G (Table S2†), implying that with 6 G nucleotides, a stable G4 conformation was adopted. This was supported by ^1^H-NMR (Fig. 4D). The intensity of the Hoogsteen imino peak, with a chemical shift in the range of 10.0 to 11.5 ppm, gradually increased for O1′-6G compared to O1′-6G and slightly reduced for O1′-7G and O1′-8G. Weak bands with molecular weights between the monomer and tetramer forms were observed in the PAGE images of O1′-6G, O1′-7G, and O1′-8G (Fig. S9†), indicating the presence of non-tetramer G-quadruplex conformations.^41^–^43^ These results also suggest that the emission intensity of O1-DTPE, enhanced by TP-G4, could be used as a parameter to examine the stability of the G-quadruplex structure.

When different numbers of G nucleotides were attached at the 3′ end of O1′, TP-G4 hybridization with O1″-DTPE showed an increasing fluorescence signal with increasing numbers of G nucleotides (Fig. S10†). However, the fluorescence intensity was not significant until 5G. From these experiments, we conclude that the G-quadruplex is less stable when the G nucleotides are at the 3′ end of the oligonucleotide than at the 5′ end.

### Emission modulation using i-motif as the clawed scaffold

**Fig. 6:**
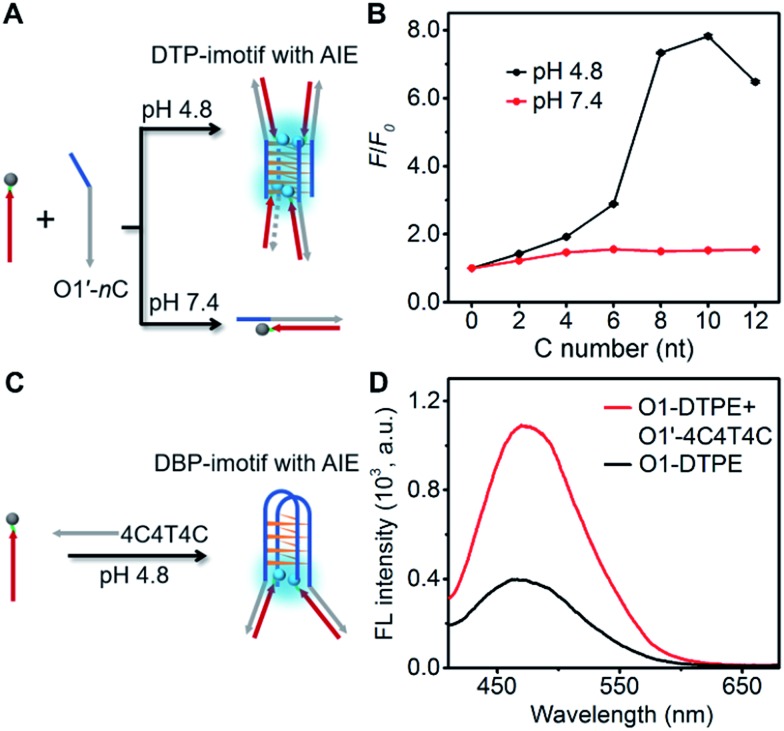
(A) Schematic illustration of the DTP-imotif structure adopted at pH 4.8 and the double-stranded structure formed at pH 7.4. (B) Ratios of fluorescent peak intensity of 1.0 µM O1-DTPE after and before (*F*/*F*_0_) hybridization with O1′-*n*C (*n* = 0 to 12) in pH 4.8 TAE reaction buffer and in pH 7.4 TE reaction buffer at 25 °C. (C) Schematic illustration of the DBP-imotif structure. (D) Fluorescence spectra of 1.0 µM O1-DTPE and the mixture of 1.0 µM O1-DTPE and O1′-4C4T4C in pH 4.8 TAE reaction buffer at 25 °C.

To develop more candidates for emission modulation of TPE, we designed a tetramolecular i-motif structure as the clawed scaffold. The double-stranded tetramolecular i-motif (DTP-imotif) should form when O1′ has a number of C nucleotides at the 5′ end in a buffer of low pH, whereas at neutral pH, a duplex should be formed (Fig. 6A).^31^ In 10 mM Tris–acetate pH 4.8, 100 mM NaCl, 10 mM MgCl_2_ and 1 mM sodium EDTA, the intensity of the positive peak at 288 nm in the CD spectrum gradually increased with the number of C nucleotides linked to O1′ (Fig. S11†). CD melting curves showed a second melting transition, presumably due to the stable i-motif, when O1′ was linked to 8 C nucleotides (Fig. S12†). When O1-DTPE was mixed with O1′-*n*C at pH 4.8, the fluorescence increased with the increasing number of C nucleotides and reached a maximum value at 10 C nucleotides (Fig. 6B). There was little difference in fluorescence at pH 7.4, demonstrating that TPE emission was dependent on i-motif formation. Similarly, O1″-DTPE also showed the fluorescence signal in the presence of i-motif-forming strands (Fig. S13†). Another clawed scaffold was constructed with O1′-4C4T4C at pH 4.8. This oligonucleotide formed a bimolecular i-motif (Fig. 6C). Upon the hybridization of O1-DTPE to this scaffold to form the double-stranded bimolecular i-motif (DBP-imotif), and the fluorescence intensity was increased by 2.7-fold over the baseline (Fig. 6D).

### Design of the AIE biosensing strategy

**Fig. 7:**
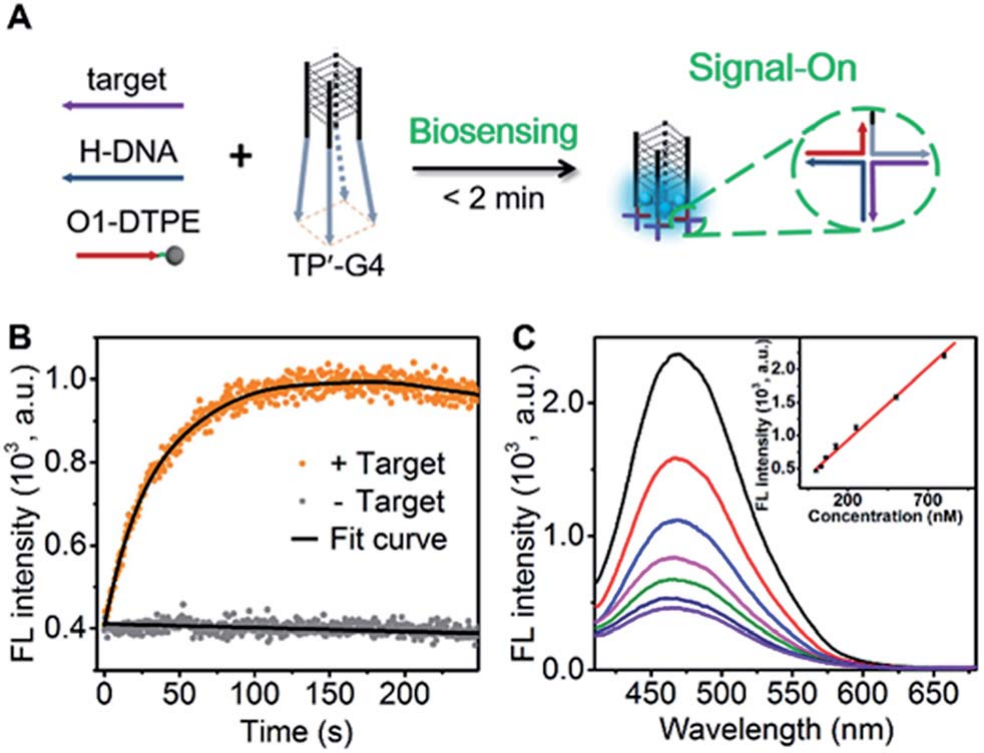
(A) Schematic illustration of the DNA detection strategy that relies on the self-assembly of four-way junctions. (B) Time-dependent fluorescence signal for 1.0 µM O1-DTPE, 1.0 µM H-DNA, and 1.0 µM TP’-G4-5 bp in the presence and absence of target DNA in TE reaction buffer at 25 °C. (C) Fluorescence spectra of 1.0 µM O1-DTPE after mixing with 1.0 µM H-DNA and TP’-G4 in the presence of target DNA at different concentrations for 1.5 min in TE reaction buffer at 25 °C. Inset is the calibration curve.

To demonstrate the utility of the TP-G4-based molecular scaffold, we designed a simple DNA assay. As shown in Fig. 7A, the TP’-G4 limbs are complementary to a region of the target, as is a region of helper DNA (H-DNA). O1-DTPE can hybridize to a region of H-DNA and to a region of the TP’-G4 limb. The resulting four-way junctions could bring multiple O1-DTPEs close to G4 and thus the emission of TPE is expected to be induced. The sequences of the strands used in this design are listed in Table S1.† The formation of a four-way junction on the limbs of TP’-G4 was verified by native PAGE. In the presence of target DNA, a band migrating more slowly than TP’-G4 was observed (Fig. S14†). Stopped-flow fluorescence measurements were also performed to monitor the assembly of the four-way junction formed by O1 labelled with FAM, H-DNA, and TP’-G4 labeled with carboxytetramethylrhodamine (TAMRA). Quenching of FAM fluorescence was observed within 100 seconds (Fig. S15†).

The proposed detection strategy resulted in the rapid increase in fluorescence within the maximum signal obtained within 2 minutes (Fig. 7B). Importantly, there is no need for separation. With the optimized number of base pairs between O1-DTPE and TP’-G4 (Fig. S16†), the light emission signal showed good linearity as a function of target DNA concentration in the range of 32.5 nM to 800 nM (Fig. 7C). Thus, the molecular scaffold with Oligo-AIEgen probes has great potential to be developed into a rapid bioassay for sequence-specific detection of DNA.

## Discussion

For AIEgens, light emission can be generated due to the restriction of intramolecular motions when they are exposed to external stimuli. The controllable assembly of AIEgens is able to provide a new opportunity for precise manipulation of the fluorescence signal transduction. In this study, we prepared four-stranded DNA G-quadruplexes and tetra- and bimolecular i-motifs as clawed scaffolds for the localized assembly and modulation of AIEgen emission. Unlike previous reports in which AIEgens were linked to oligonucleotides with short alkyl chains and the fluorescence increased upon formation of a double-stranded structure,^36^,^37^ the 15 nt AIEgen prepared here yielded no fluorescence signal when hybridized to a complementary oligonucleotide. In our Oligo-AIEgen design, the oligonucleotides were linked to TPE through a longer, more flexible chain that allowed the TPE moiety to rotate freely. An intense signal was only observed from O1-DTPE in the presence of O1′-6G, which formed a parallel-stranded tetramolecular G-quadruplex; no signal was observed in the presence of O1′-6A, O1′-6C or O1′-6T. Moreover, we designed a G-quadruplex with the single limb O1′-mG4. Upon the addition of O1-DTPE, a fluorescence enhancement was observed (Fig. S17†), which should be attributed to the association between the G-quadruplex structure of O1′-mG4 and TPE molecules because O1′-mG4 hybridized only one O1-DTPE strand at the single limb.^44^,^45^ When the G-quadruplex was destabilized in the presence of Li^+^ ions, the AIE signal was switched off. Furthermore, DNA G-quadruplex structures formed by O1-6G and 15T-6G, which have limbs that are not complementary to O1, were not able to induce the fluorescence of O1-DTPE. These data indicate that both the G-quadruplex scaffold and specific hybridization to the clawed scaffold limbs are necessary to manipulate the fluorescence signal of TPE, the iconic AIEgen.

To manipulate the light emission of TPE, single-stranded spacers were inserted between the parallel G-quadruplex conformation and limbs. Since steric hindrance and electrostatic repulsion exist between four limbs of the G4, the four limbs form a rectangular pyramid structure. With an increase of spacer length, the angle increased and the distance between each TPE molecule became larger, resulting in a decreased degree of AIEgen interactions and lower intensity of TPE signals (Fig. 1C). The influence of distance from the core was further demonstrated by analysis of fluorescence from O1″-DTPE in the presence of DTP(10)-G4 and DTP(15)-G4. Presumably due to less effective interactions among TPE molecules at a lower position of the rectangular pyramid, there was less signal from DTP(15)-G4 than DTP(10)-G4. Therefore, the light emission of TPE could be precisely regulated by controlling the distance between the scaffold’s G4 core and the AIEgen.

The stability of the parallel G-quadruplex conformation is dependent on the number of G quartets.^27^,^30^,^33^,^46^ Thus the TPE light emission could theoretically be controlled by altering the G quartet number. Experimental results showed that O1-DTPE fluorescence increased and then plateaued as the thermodynamic stability of the G4 increased. For O1′-7G and O1′-8G, the signal was suppressed. This is likely due to the generation of bimolecular G-quadruplex structures, which do not effectively induce AIEgen interactions. We also demonstrated control of the light emission process by altering the number of C nucleotides in the i-motif scaffold.

Previously, AIE aggregates were produced by the reactions between target analytes and a functionalized AIEgen,^11^,^13^ for example by enzymatic cleavage. The lack of control of aggregation could result in false positive signals. AIEgens can also be linked through target analytes to form aggregates;^2^,^15^ however, functionalized AIEgens must be carefully designed and sophisticated synthesis is required. In this work, AIE assembly was induced by the predictable structure of G4 and by an i-motif scaffold. We demonstrated the potential of the TP-G4 system as a biosensor in an assay in which target DNA was required to bring the Oligo-AIEgen into proximity of the G4 core to induce fluorescence. A signal was generated rapidly and was linear in the range of 32.5 nM to 800 nM. The introduction of DNA circles with AIE-based TP-G4 is expected to further improve the sensitivity of the probe. Moreover, functionalization with targeted-aptamers could not only enable detection of various proteins or small molecules but also provide potential applications for bioimaging due to the unique properties of AIEgens.^47^

## Conclusions

This work demonstrated the use of molecular scaffolds for localized assembly and modulation of light emission of Oligo-AIEgens. Regulation is based on the controllable assembly of AIEgens in the confined space within the four limbs of TP-G4 or the i-motif, which leads to the “on” state of the probe. The signal output has been successfully modulated by altering the distance between TPE and the core of the scaffold, as well as by altering the stability of the core. The clawed scaffold-based system was further used to form a sequence-specific DNA detection assay, demonstrating that AIE is a promising mode of signal transduction in the design of biosensors.

## Experimental

### Reagents

Tris(hydroxymethyl)aminomethane (Tris), 1,2-bis[4-(azidomethyl)phenyl]-1,2-diphenylethene (TPE-N_3_), and dibenzocyclooctyne-sulfo-*N*-hydroxysuccinimidyl ester (DBCO-sulfo-NHS) were purchased from Sigma-Aldrich Inc. (St. Louis, MO, USA). Gel electrophoresis loading buffer was from Solarbio. Co., Ltd. (Beijing, China). UltraPowerTM dye was from Bioteke, Co., Ltd. (Beijing, China). TE buffer (pH 7.4) was prepared using 10 mM Tris–HCl and 1 mM sodium EDTA. TAE buffer (pH 4.8) contained 10 mM Tris–acetate and 1 mM sodium EDTA. The TE and TAE reaction buffers were prepared by mixing 100 mM NaCl and 10 mM MgCl_2_ in these buffers, respectively. Li^+^ buffer (pH 7.4) contained 10 mM Tris–HCl, pH 7.4, 100 mM LiCl, 10 mM MgCl_2_, and 1 mM sodium EDTA. All aqueous solutions were prepared using ultrapure water (≥18.2 MΩ cm, Milli-Q). Oligonucleotide-alkyl chain-grafted DBCO (O1-DBCO, O2-DBCO and O1″-DBCO) was synthesized by Takara Bio Inc. (Dalian, China). Other DNA sequences were obtained from Sangon Biotech Co., Ltd. (Shanghai, China). These DNA sequences are listed in Table S1.† Prior to use, the DNA oligonucleotides were dissolved in TE buffer (pH 7.4) or TAE buffer (pH 4.8) at 200 µM strand concentration. To form the DNA G-quadruplex structures, DNA oligonucleotides were diluted into the reaction buffer (pH 7.4), heated to 95 °C, cooled gradually to room temperature (0.6 °C min^–1^), and stored at 4 °C for two days prior to use.

### Apparatus

Absorption spectra were recorded on a Cary 300 UV-VIS Spectrophotometer (Agilent). Gel electrophoresis was performed on a mini-PROTEAN system (Bio-Rad) and imaged on a Bio-Rad ChemDoc XRS. Fluorescence spectra were measured on an F-7000 spectrofluorophotometer (Hitachi) with a TC125 temperature control (Quantum Northwest). The dynamic light scanning (DLS) measurements of TPE-N_3_ in DMSO/H_2_O (v/v, 1/399) and O1-DTPE in H_2_O were performed on a 90Plus Particle Size Analyzer (Brookhaven Instruments Corporation). Circular dichroism (CD) measurements were obtained at 25 °C using 1 mm path length cuvettes (Hellma) on a Chirascan CD spectrometer (Applied Photophysics). Stopped-flow spectroscopic analyses were performed on a Chirascan spectrometer equipped with a stopped-flow accessory. The ^1^H spectra were collected on an Avance 850 MHz spectrometer (Bruker) equipped with a triple resonance 5 mm HCN-cryoprobe at 25 °C.

### Synthesis of Oligo-AIEgens

The oligonucleotide-grafted AIE fluorogens (Oligo-AIEgens), O1-DTPE, O2-DTPE, and O1″-DTPE, were synthesized by mixing 0.5 mL of O1-DBCO, O2-DBCO, or O1″-DBCO (200 µM) and 0.5 mL of 8.0 mM TPE-N_3_ in DMSO with gentle stirring at room temperature overnight. The products were purified by PAGE. Quantitative analysis by ultraviolet (UV) spectroscopy revealed a coupling yield of 30%.

### ^1^H-NMR experiments

The G-quadruplex structures were formed in 10 mM phosphate buffer pH 7.4, containing 100 mM Na^+^ and 10 mM Mg^2+^, using the annealing procedure described in the Reagents section. Spectra were recorded with a spectral width of 18 750 Hz, a data point of 16 K, transients of 1024, a relaxation delay of 1.0 s, and a 90° pulse at 11.75 µs. Water suppression was accomplished using the WATERGATE W5 pulse sequence with gradients.^48^

### Fluorescence measurements

The excitation wavelength was set to 350 nm, and the emission spectra were scanned from 410 nm to 680 nm for Oligo-AIEgens. The excitation wavelengths were set to 350, 495, and 548 nm to measure the emission intensity of O1-AMCA, O1-FAM, and O1-Cy3 at 470, 515, and 560 nm, respectively. As in typical fluorescence measurements, Oligo-AIEgen (1.0 µM) was mixed with an equal molar amount of DNA strand in reaction buffer at room temperature. Unless noted, all fluorescence data were recorded at 25 °C.

### CD analysis

For the CD spectra, the scan speed was 1 nm s^–1^. Data were collected from 350 nm to 200 nm. The thermal denaturation experiments were set to scan from 25 to 95 °C with a temperature step of 1 °C per 0.5 min (tolerance 0.10 °C) with a TC125 temperature control.^25^

### Stopped-flow fluorescence analysis

A mixture of 2.0 µM H-DNA and TP-G4-TAMRA was injected into channel A, and a mixture of 2.0 µM target DNA and O1-FAM was injected into channel B. Stop-flow measurements were performed by monitoring the time-dependent fluorescence with an excitation wavelength of 494 nm and emission intensity of 520 nm.

### Biosensing

A rapid homogeneous biosensing strategy with the designed clawed scaffold of AIE was performed by mixing 1.0 µM O1-DTPE, 1.0 µM helper DNA (H-DNA), 1.0 µM TP’-G4 and different concentrations of target DNA in 200 µL pH 7.4 reaction buffer. After 1.5 minutes, the fluorescence was measured. For real-time fluorescence monitoring, the reaction mixture contained 1.0 µM O1-DTPE, H-DNA, TP’-G4, and target DNA in 200 µL pH 7.4 reaction buffer. The fluorescence was continuously measured over time with excitation at 350 nm and an emission wavelength of 470 nm.

## Conflicts of interest

There are no conflicts to declare.

## Supplementary Material

Supplementary information
