# Supplementary material for: DNA quadruplexes as molecular scaffolds for controlled assembly of fluorogens with aggregation-induced emission
Source: Chem Sci. 2018 Jan 29;9(9):2559–66. doi: 10.1039/c8sc00001h (PMC5911820; doi:10.1039/c8sc00001h)
Supplement: Supplementary file 1 [file SC-009-C8SC00001H-s001.pdf]

Electronic Supplementary Material (ESI) for Chemical Science.
This journal is © The Royal Society of Chemistry 2018

Electronic Supplementary Information

DNA quadruplexes as molecular scaffolds for controlled assembly of fluorogens with aggregation-induced emission

Longyi Zhu,[a] Jun Zhou,[a] Guohua Xu,[b] Conggang Li,[b] Pinghua Ling,[a] Bin Liu,*[c] Huangxian Ju*[a] and Jianping Lei*[a]

[a]State Key Laboratory of Analytical Chemistry for Life Science, School of Chemistry and Chemical Engineering, Nanjing University, Nanjing 210023, China.

[b]State Key Laboratory of Magnetic Resonance and Atomic and Molecular Physics, Wuhan Institute of Physics and Mathematics, Chinese Academy of Sciences, Wuhan 430071, China.

[c]Department of Chemical and Biomolecular Engineering, National University of Singapore, 4 Science Drive 4, Singapore 117585, Singapore.

*Address correspondence to J.P.L. (email: jpl@nju.edu.cn), H.X.J. (email: hxju@nju.edu.cn) or to B.L. (email: cheliub@nus.edu.sg).

Supporting Figures

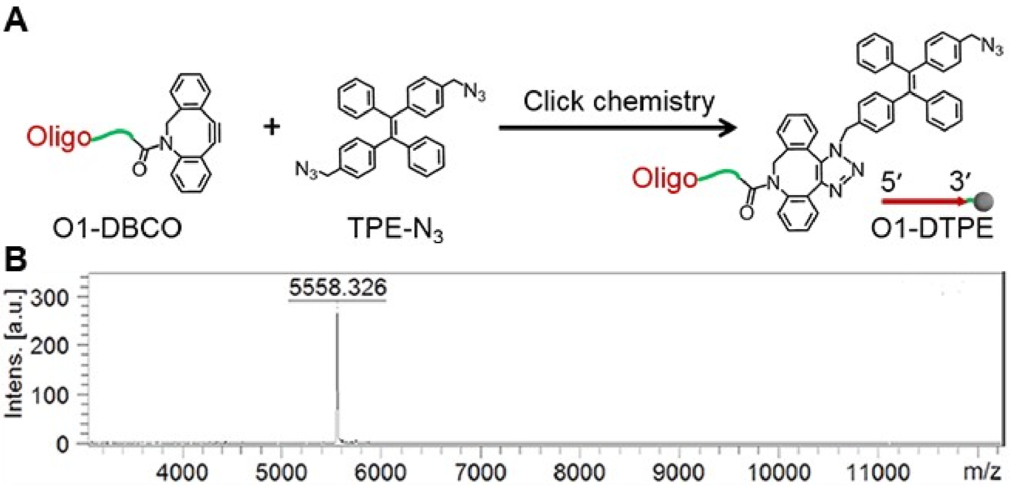

Fig. S1 (A) Synthetic scheme of O1-DTPE, (B) TOF-MS of O1-DTPE.

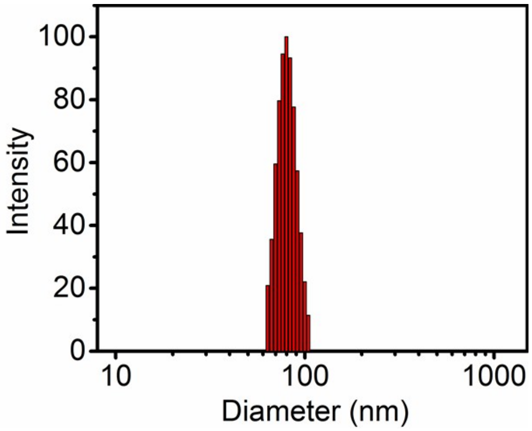

Fig. S2 Dynamic light scattering analysis of 1.0 μM TPE-N_3 in DMSO/H_2O (v/v, 1/399). No signal was observed for O1-DTPE in H_2O at 25 ^oC.

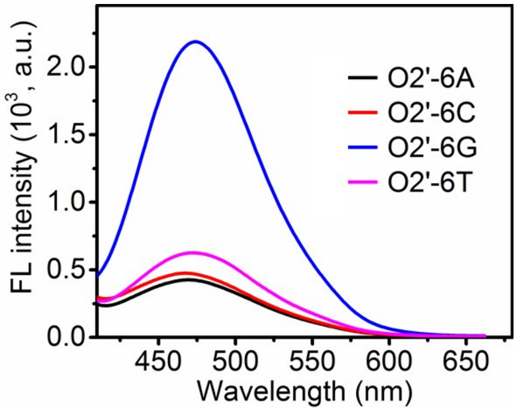

Fig. S3 Fluorescent spectra of 1.0 μM O2-DTPE in presence of indicated strands.

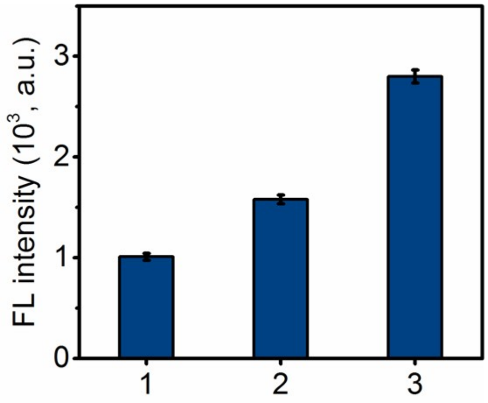

Fig. S4 Fluorescent intensity of mixture of 1.0 μM O1′-6G with (1) 0.25 μM, (2) 0.5 μM and (3) 1.0 μM O1-DTPE.

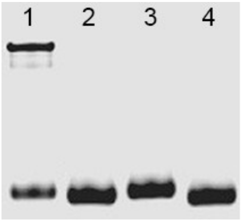

Fig. S5 PAGE results of 10 μM O1-DTPE hybridized with (1) O1′-6G, (2) O1′-6C, (3) O1′-6T and (4) O1′-6A.

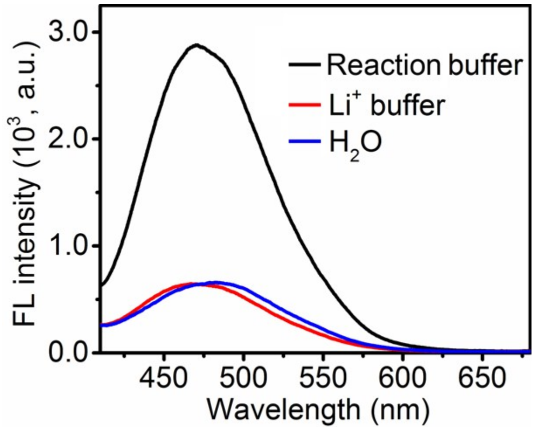

Fig. S6 Fluorescent spectra of 1.0 μM O1-DTPE hybridized with O1′-6G (1.0 μM) in TE reaction buffer (10 mM TE, pH 7.4, 100 mM NaCl, 10 mM $MgCl_2$), Li^+ buffer, and H_2O.

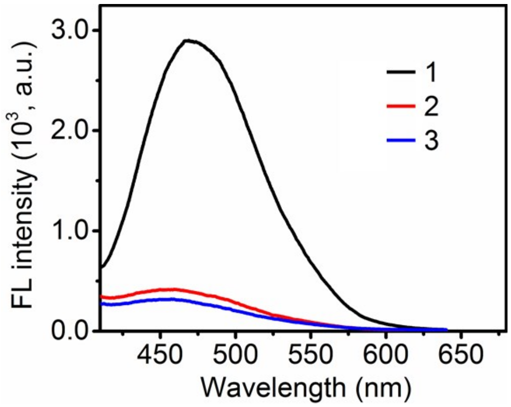

Fig. S7 Fluorescent spectra of 1.0 μM O1-DTPE mixed with (1) O1′-6G, (2) O1-6G, and (3) 15T-6G.

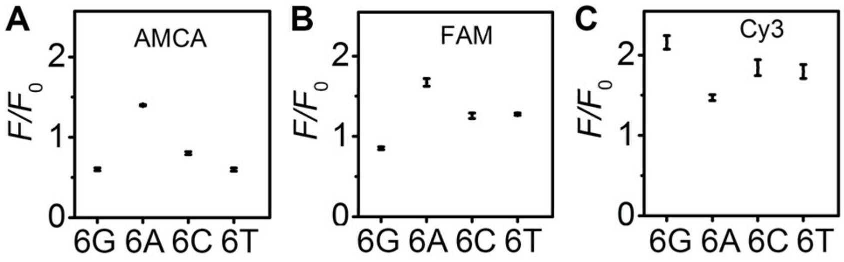

Fig. S8 Ratios of fluorescent peak intensity of 1.0 μM (A) O1-AMCA, (B) O1-FAM, and (C) O1-Cy3 after to before (F/F_0) hybridization with 1.0 μM O1′ with 6G, 6A, 6C, and 6T.

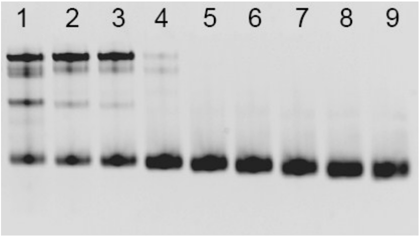

Fig. S9 PAGE results of O1-DTPE hybridized with (1) O1′-8G, (2) O1′-7G, (3) O1′-6G, (4) O1′-5G, (5) O1′-4G, (6), O1′-3G, (7) O1′-2G, (8) O1′-1G, and (9) O1′.

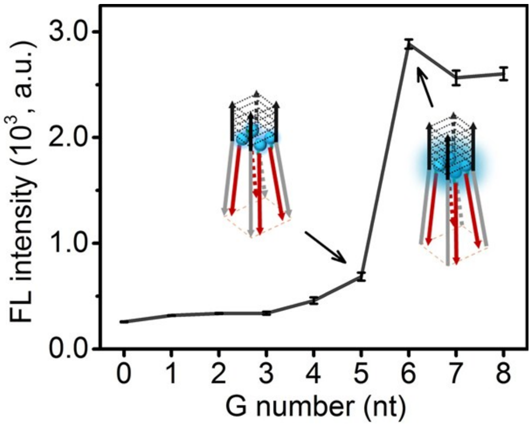

Fig. S10 Fluorescent intensity (FL) of 1.0 μM O1″-DTPE hybridized with O1′ bearing 0 to 8 Gs at 3′ end.

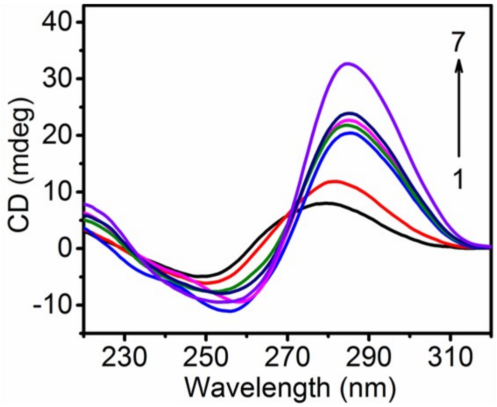

Fig. S11 CD spectra of 20 μM (1) O1, (2) O1′-2C, (3) O1′-4C, (4) O1′-8C, (5) O1′-6C, (6) O1′-10C and (7) O1′-12C in pH 4.8 TAE reaction buffer (10 mM Tris-acetate, 100 mM NaCl, 10 mM $MgCl_2$, 1 mM sodium EDTA).

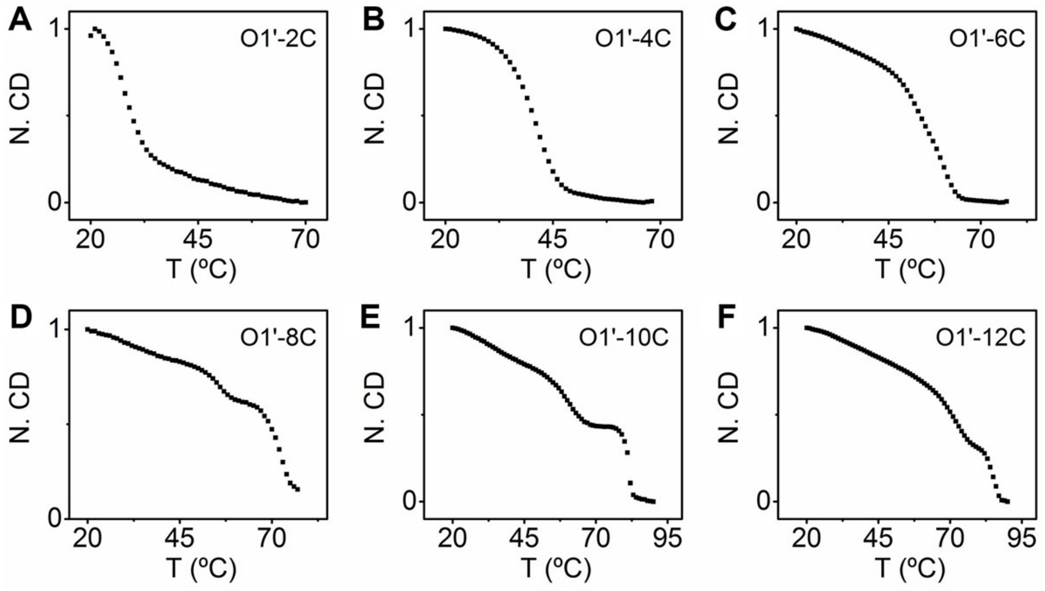

Fig. S12 Normalized CD (N. CD) melting curves monitored at 285 nm of 20.0 μM O1′ bearing different numbers of Cs (O1′-*n*C, *n* = 2 to 12); temperature was increased at a rate of 2.0 ^{o}C/min. Data are normalized to the highest CD value at 285 nm.

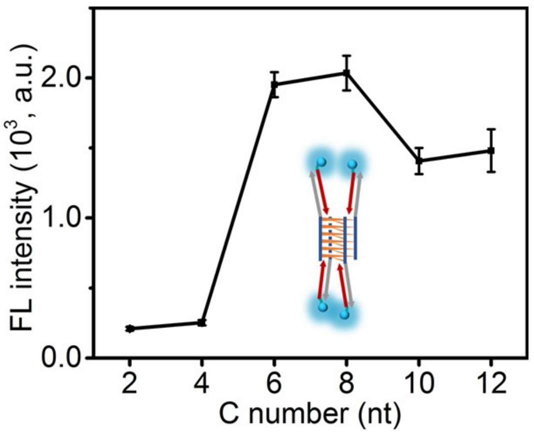

Fig. S13 Fluorescence intensity of 1.0 μM O1″-DTPE hybridized with O1′-*n*C (*n* = 0 to 12) in pH 4.8 TAE reaction buffer.

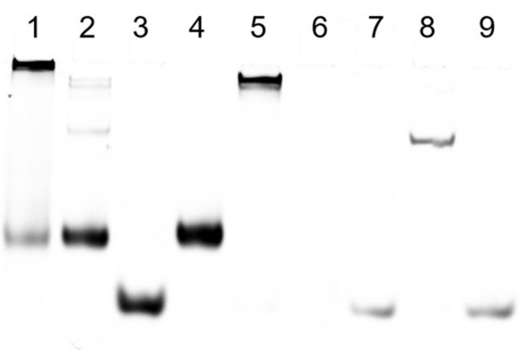

Fig. S14 PAGE results of (1) target DNA + TP′-G4-5bp + H-DNA + O1-DTPE, (2) TP′-G4-5bp + H-DNA + O1-DTPE, (3) TP′-G4-5bp + H-DNA, (4) H-DNA + O1-DTPE, (5) TP′-G4-5bp + O1-DTPE, (6) O1-DTPE, (7) H-DNA, (8) TP′-G4-5bp, (9) target DNA.

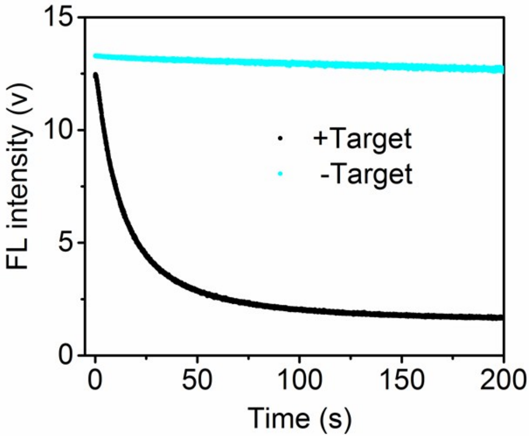

Fig. S15 Stopped-flow fluorescence over time for the mixtures of 1.0 μM O1-FAM, H-DNA, and TP′-G4-TAMRA in presence and absence of target DNA.

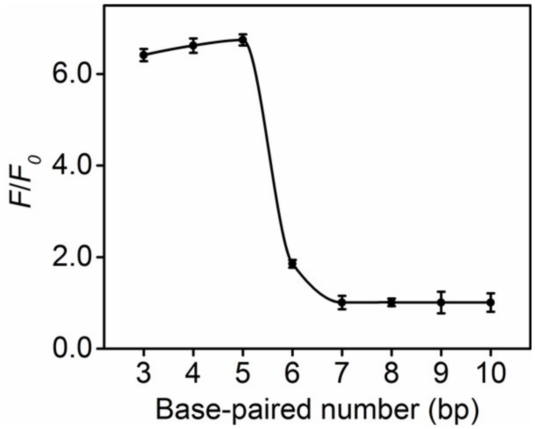

Fig. S16 Fluorescence intensity of a mixture of 1.0 μM H-DNA, target DNA, O1-DTPE, and 16-nt limbs of TP′-G4 with different numbers of base pairs (TP′-G4-*n*bp, *n* = 3 to 10) in pH 7.4 reaction buffer.

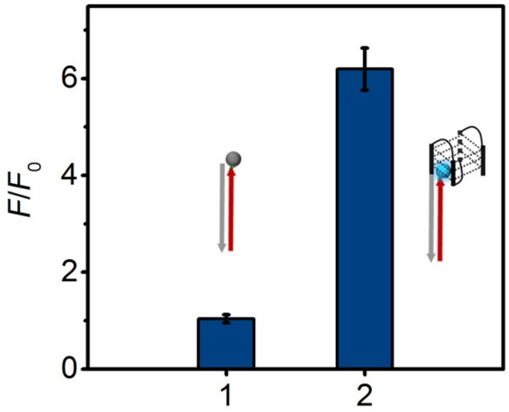

Fig. S17 Ratios of fluorescent peak intensity of 1.0 μM O1-DTPE after to before (F/F_0) hybridization with (1) O1′and (2) O1′-mG4 in TE reaction buffer.

Supporting Tables

Table. S1 Oligonucleotide sequences used in the experiments.

Name	Sequences (from 5′ to 3′ end)
O1-DBCO	AGTTGGAGACGTAAG-$(CH_2)_6NHCO(CH_2)_4$-DBCO
O2-DBCO	CCTACGTCTCCAACTAACTTACGGCCCTCATTCAATACCCTACG-$(CH_2)_6NHCO(CH_2)_4$-DBCO
O1″-DBCO	DBCO-$(CH_2)_4CONH(CH_2)_6$-AGTTGGAGACGTAAG
O1′	CTTACGTCTCCAACT
O1′-1G	GCTTACGTCTCCAACT
O1′-2G	GGCTTACGTCTCCAACT
O1′-3G	GGGCTTACGTCTCCAACT
O1′-4G	GGGGCTTACGTCTCCAACT
O1′-5G	GGGGGCTTACGTCTCCAACT
O1′-6G	GGGGGGCTTACGTCTCCAACT
O1′-7G	GGGGGGGCTTACGTCTCCAACT
O1′-8G	GGGGGGGGCTTACGTCTCCAACT
O1′-6A	AAAAAACTTACGTCTCCAACT
O1′-6T	TTTTTTCTTACGTCTCCAACT
O1′-2C	CCCTTACGTCTCCAACT
O1′-4C	CCCCCTTACGTCTCCAACT
O1′-6C	CCCCCCCTTACGTCTCCAACT
O1′-8C	CCCCCCCCCTTACGTCTCCAACT
O1′-10C	CCCCCCCCCCCTTACGTCTCCAACT
O1′-12C	CCCCCCCCCCCCCTTACGTCTCCAACT
O1′-5A-6G	GGGGGGAAAAACTTACGTCTCCAACT
O1′-10A-6G	GGGGGGAAAAAAAAAACTTACGTCTCCAACT
O1′-15A-6G	GGGGGGAAAAAAAAAAAAAAACTTACGTCTCCAACT
O1′-20A-6G	GGGGGGAAAAAAAAAAAAAAAAAAAACTTACGTCTCCAACT
O1′-5T-6G	GGGGGGTTTTTCTTACGTCTCCAACT
O1′-10T-6G	GGGGGGTTTTTTTTTTCTTACGTCTCCAACT
O1′-15T-6G	GGGGGGTTTTTTTTTTTTTTTCTTACGTCTCCAACT

O1′-20T-6G	GGGGGGTTTTTTTTTTTTTTTTTTTTCTTACGTCTCCAACT
O1′-6G-5A	GGGGGGCTTACGTCTCCAACTAAAAA
O1′-6G-10A	GGGGGGCTTACGTCTCCAACTAAAAAAAAAA
O1′-6G-15A	GGGGGGCTTACGTCTCCAACTAAAAAAAAAAAAAAA
O1′-6G-20A	GGGGGGCTTACGTCTCCAACTAAAAAAAAAAAAAAAAAAAA
O1′-6G-5T	GGGGGGCTTACGTCTCCAACTTTTTT
O1′-6G-10T	GGGGGGCTTACGTCTCCAACTTTTTTTTTTT
O1′-6G-15T	GGGGGGCTTACGTCTCCAACTTTTTTTTTTTTTTTT
O1′-6G-20T	GGGGGGCTTACGTCTCCAACTTTTTTTTTTTTTTTTTTTTT
O2′-6A	AAAAAACGTAGGGTATTGAATGAGGGCCGTAAGTTAGTTGGAGACGTAGG
O2′-6C	CCCCCCCGTAGGGTATTGAATGAGGGCCGTAAGTTAGTTGGAGACGTAGG
O2′-6T	TTTTTTCGTAGGGTATTGAATGAGGGCCGTAAGTTAGTTGGAGACGTAGG
O2′-6G	GGGGGGCGTAGGGTATTGAATGAGGGCCGTAAGTTAGTTGGAGACGTAGG
O1-6G	GGGGGGAGTTGGAGACGTAAG
15T-6G	GGGGGGTTTTTTTTTTTTTTT
O1-AMCA	AGTTGGAGACGTAAG-AMCA
O1-FAM	AGTTGGAGACGTAAG-FAM
O1-Cy3	AGTTGGAGACGTAAG-Cy3
1G-O1′	CTTACGTCTCCAACTG
2G-O1′	CTTACGTCTCCAACTGG
3G-O1′	CTTACGTCTCCAACTGGG
4G-O1′	CTTACGTCTCCAACTGGGG
5G-O1′	CTTACGTCTCCAACTGGGGG
6G-O1′	CTTACGTCTCCAACTGGGGGG
7G-O1′	CTTACGTCTCCAACTGGGGGGG
8G-O1′	CTTACGTCTCCAACTGGGGGGGG
O1′-4C4T4C	CCCCTTTTCCCCTCTTACGTCTCCAACT
TP(10)-G4	GGGGGGGTCTCCAACTAAAAA
TP′-G4-3bp	GGGGGGTCAATACTACCTCA
TP′-G4-4bp	GGGGGGTCAAATACTACCTCA
TP′-G4-5bp	GGGGGGTCAACATACTACCTCA
TP′-G4-6bp	GGGGGGTCAACCATACTACCTCA

TP′-G4-7bp	GGGGGGTCAACCTATACTACCTCA
TP′-G4-8bp	GGGGGGTCAACCTCATACTACCTCA
TP′-G4-9bp	GGGGGGTCAACCTCTATACTACCTCA
TP′-G4-10bp	GGGGGGTCAACCTCTGATACTACCTCA
H-DNA	AACTATACAAGCTCTGCATTC
Target	TGAGGTAGTATCTTGTATAGTT
TP′-G4-TAMARA	GGGGGGT(TAMRA)CAACATACTACCTCA
O1′-mG4	GGGTGGGTGGGTGGGTCTTACGTCTCCAACT

The blue bases are designed to form the nanostructure core or equivalent region in controls. FAM is 6-carboxyfluorescein, AMCA is aminomethylcoumarin, and Cy3 is cyanine 3.

Table. S2 CD melting temperatures (T_ms) of O1′ bearing different numbers of Gs at the 5′ end.

DNA strands	O1′	O1′-1G	O1′-2G	O1′-3G	O1′-4G	O1′-5G	O1′-6G	O1′-7G	O1′-8G
T_m / ^oC	-	-	-	< 20	44	49	53	54	54
